# Open-hardware design and characterization of an electrostatic aerosol precipitator

**DOI:** 10.1016/j.ohx.2022.e00266

**Published:** 2022-01-10

**Authors:** Sabin Kasparoglu, Timothy P. Wright, Markus D. Petters

**Affiliations:** NC State University, Department of Marine Earth and Atmospheric Sciences, Raleigh, NC 27695-8208, United States

**Keywords:** Aerosol science, Aerosol technology, Electrostatic precipitator, Dual tandem differential mobility analyzer

## Abstract

Electrostatic precipitators are devices that remove charged particles from an air stream. We present the design and characterization of an electrostatic precipitator that is intended to be incorporated into aerosol sampling equipment. Hardware and software components of the design are open, all components can be directly purchased from vendors, and the device can be assembled with standard tools. Generic components are used to allow the repurposing of parts for other uses. The computer-controlled high-voltage power supply box associated with the project can be used for other common high-voltage applications in Aerosol Science and Technology, such as data acquisition and control systems for scanning mobility particle sizers. Computational fluid dynamics simulations are used to quantify the 3D flow field. The transfer function associated with the partial transmission is characterized through modeling and experiments. The observed transfer function is unique but deviates from the ideal transfer function due to the distortion of the flow near the inlet and the outlet of the device. Singly charged particles up to 624 nm and 253 nm can be completely removed for 0.5 L min^−1^ and 1 L min^−1^, respectively. We anticipate that our device will increase the accessibility of the technique to a broader audience.

## Graphical abstract

**Figure:**
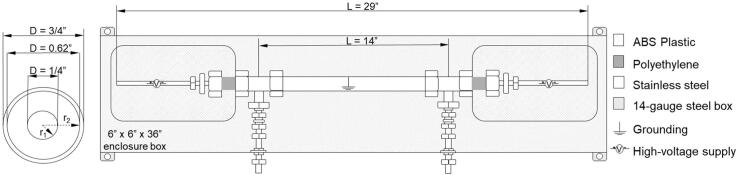

**Specifications table:** 

Hardware name	Electrostatic Aerosol Precipitator
Subject area	• Engineering and materials science • Educational tools and open source alternatives to existing infrastructure
Hardware type	● Measuring physical properties and in-lab sensors ● Field measurements and sensors ● Aerosol sampling equipment
Closest commercial analog	Cambustion Aerosol Electrostatic Precipitator
Open source license	All software is distributed under the GPL-v3 license. The design is licensed under the Attribution-NonCommercial-ShareAlike 4.0 International Public License (CC BY-NC-SA 4.0 International)
Cost of hardware	USD 1550
Source file repositories	https://doi.org/10.5281/zenodo.5295817 https://doi.org/10.5281/zenodo.5295711 https://doi.org/10.5281/zenodo.5295725

## Hardware in context

Electrostatic precipitators (EP) have been in use since the early 1900 s. In EPs, charged particles are passed between two electrodes that create an electrostatic field. The electrostatic field deflects the particle either toward or away from the electrode and deposits it on the surface. The transmission efficiency of the EP depends on the particle size, the number of elementary charges on the particles, the electric field strength, and the residence time of the particle within the field [1]. Electrostatic precipitators are commonly used to purify gases in industrial processes [2], to control indoor air pollution [3], to collect particles onto substrates for further analysis [4], [5], [6], [7], [8], [9], [10], [11], [12], [13], to control particle size evolution [14], and to remove charged particles from an air stream as part of a chain of aerosol instruments [15], [16], [17]. Commercial versions for removal of particles as part of aerosol instrumentation are available. For example, the Electrostatic Precipitator from Cambustion Ltd. (Cambridge, United Kingdom) that is used as an accessory with the centrifugal particle mass analyzer [17].

## Hardware description

This work details the design and characterization of an EP that has the purpose to remove charged particles from an air stream. The EP was developed in-house as part of the dual tandem differential mobility analyzer (DMA) [16], [18], which is used to create dimer particles for viscosity measurement (e.g. refs. [19], [20], [21], [22]). A simple DMA possesses an annulus gap between inner and outer cylinder columns where the applied electric potential drags the charged particles depending on their mobility with the sheath flow [23].

**Fig. 1:**
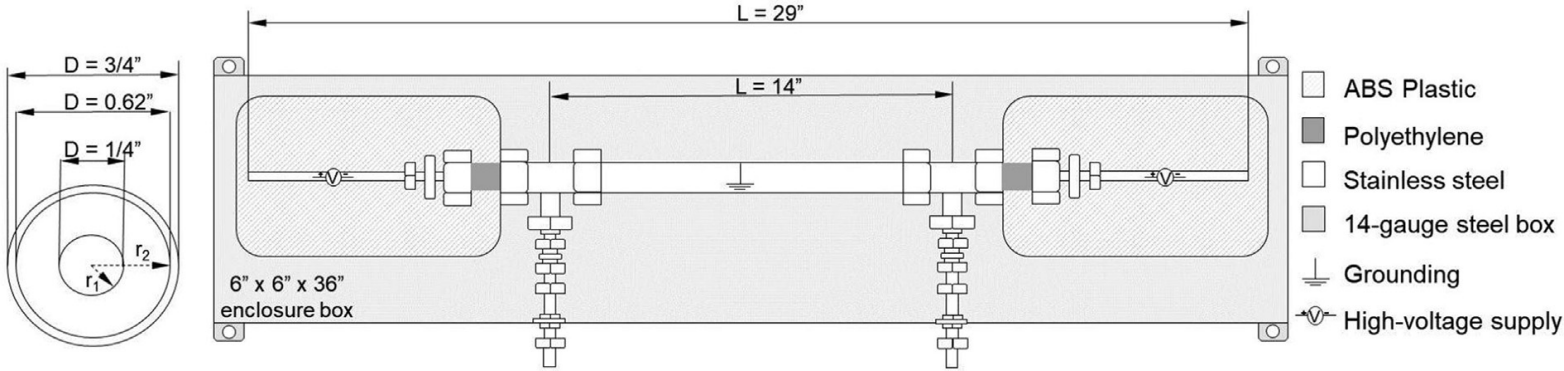
Technical drawing of the electrostatic precipitator.

Fig. 1 shows a technical drawing of the precipitator. Since the items were ordered from vendors in the United States (U.S.), the U.S. customary measurement system units are used throughout to maintain readability. Conversions from the U.S. to metric units are 1′ (one foot) = 30.48 cm and 1″ (one inch) = 2.54 cm. The design is that of a cylindrical capacitor. Sample is introduced at a 90°angle through a T-shaped tube fitting. The sample, consisting of charged particles and carrier gas, flows through the annulus gap. The outer tube is electrically grounded. The inner tube is held at a constant potential. Charged particles are deflected either toward or away from the center rod. All particles with electrical mobility exceeding a threshold value will be filtered by the device. Particles without electric charge or particles with low electrical mobility exit with air flow.

**Fig. 2:**
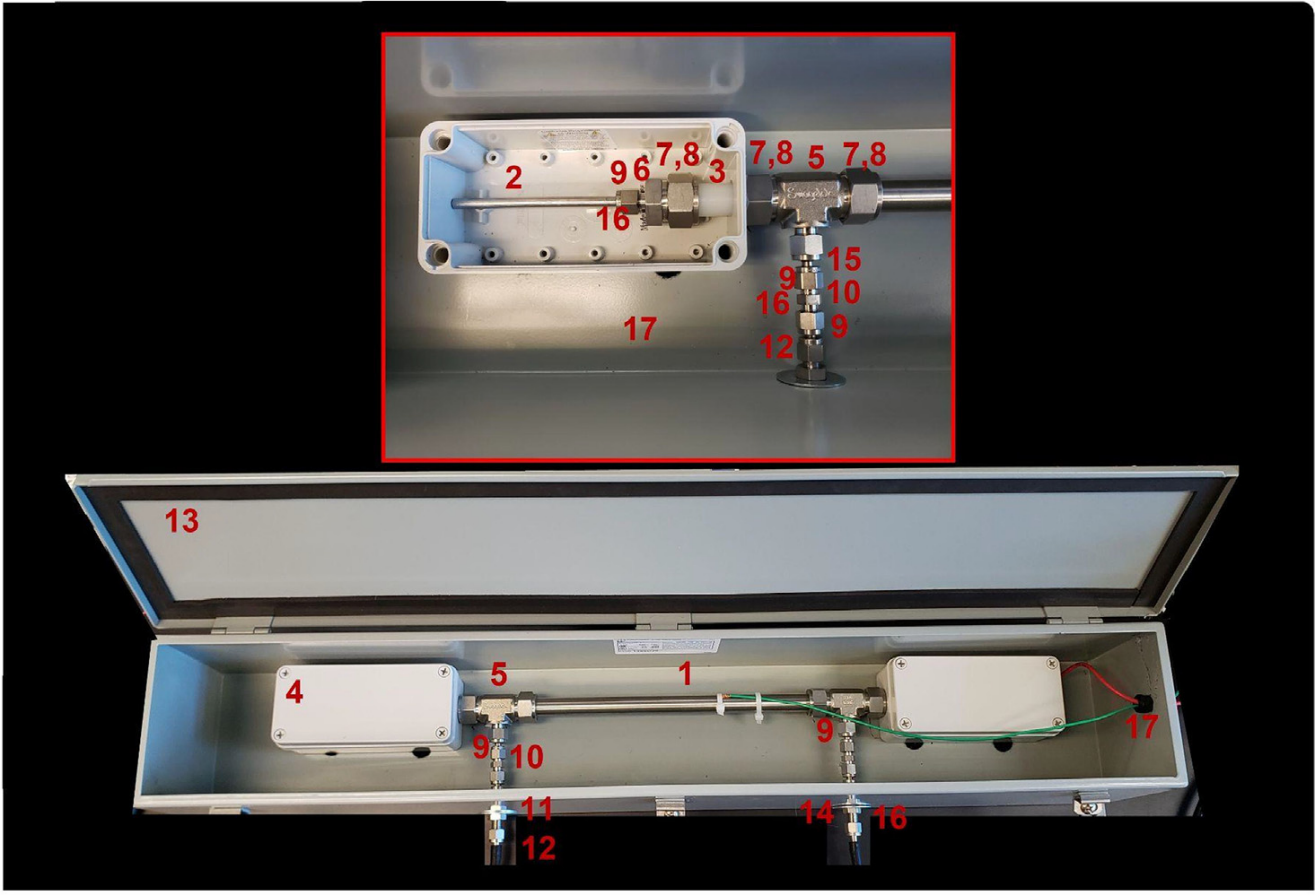
Assembled electrostatic precipitator. The numbering of the parts is given in the following instruction.

Photographs of the assembled instrument are provided in Fig. 2. The charge-carrying electrode consists of a 29″ long, 1/4″ diameter, 316L stainless steel rod. The outer conductor is a 12″ long, smooth-bore, seamless 316 stainless steel tubing with 3/4″ outer diameter and 0.065″ wall thickness. Ultra high molecular weight polyethylene tubing, 3″ long, 3/4″ outer diameter, and 1/4″ inner diameter are used to mount the inner electrode and to electrically isolate the electrode and outer wall on both sides of the device. Compression fittings (Swagelok, Solon, Ohio, U.S.A.) are used to seal the device. Stainless steel reducing union tee fittings, (3/4″ x 3/4″ x 3/8″) tube outer diameter connect the (3/4″) polyethylene tube and stainless steel tubes and provide a (3/8″) inlet and outlet mounted at a 90-degree angle relative to the streamwise flow. The outer wall is grounded. The inner electrode is connected to a high-voltage DC power supply. The inner electrode and compression fittings are unsafe to touch and are electrically isolated using enclosures made from acrylonitrile butadiene styrene (ABS) plastic. The entire device is placed in a secondary enclosure for stability and ease of transport. The (3/8″) inlet/outlet is connected to a (3/8″ to 1/4″) reducing union that passes the flow through to the outside of the secondary enclosure. Assembly of the device requires standard tools, including a power drill, a saw, and wrenches. The cost of the hardware is less than $700 (All costs are in USD).

**Fig. 3:**
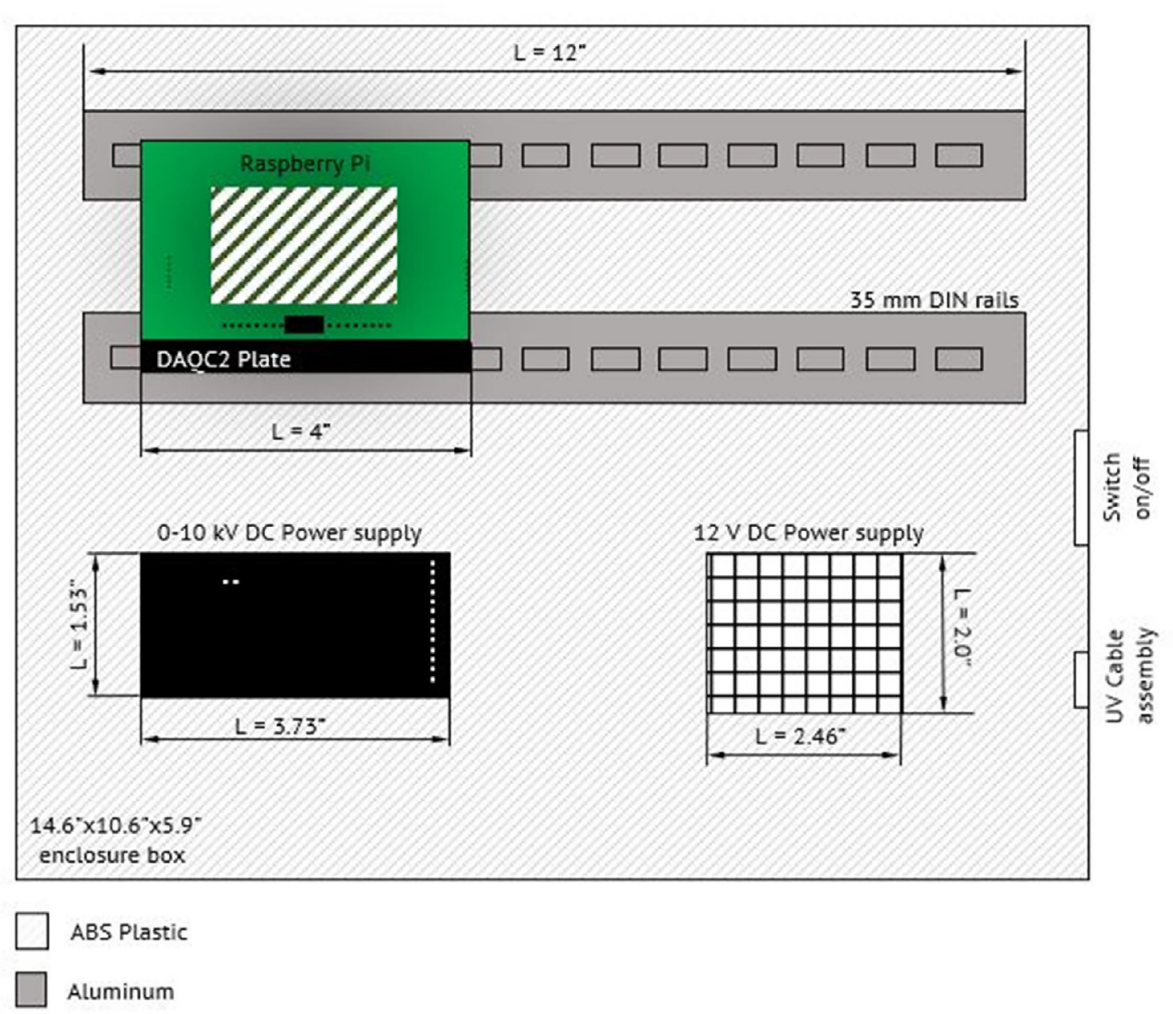
Schematic of the high-voltage power supply box.

**Fig. 4:**
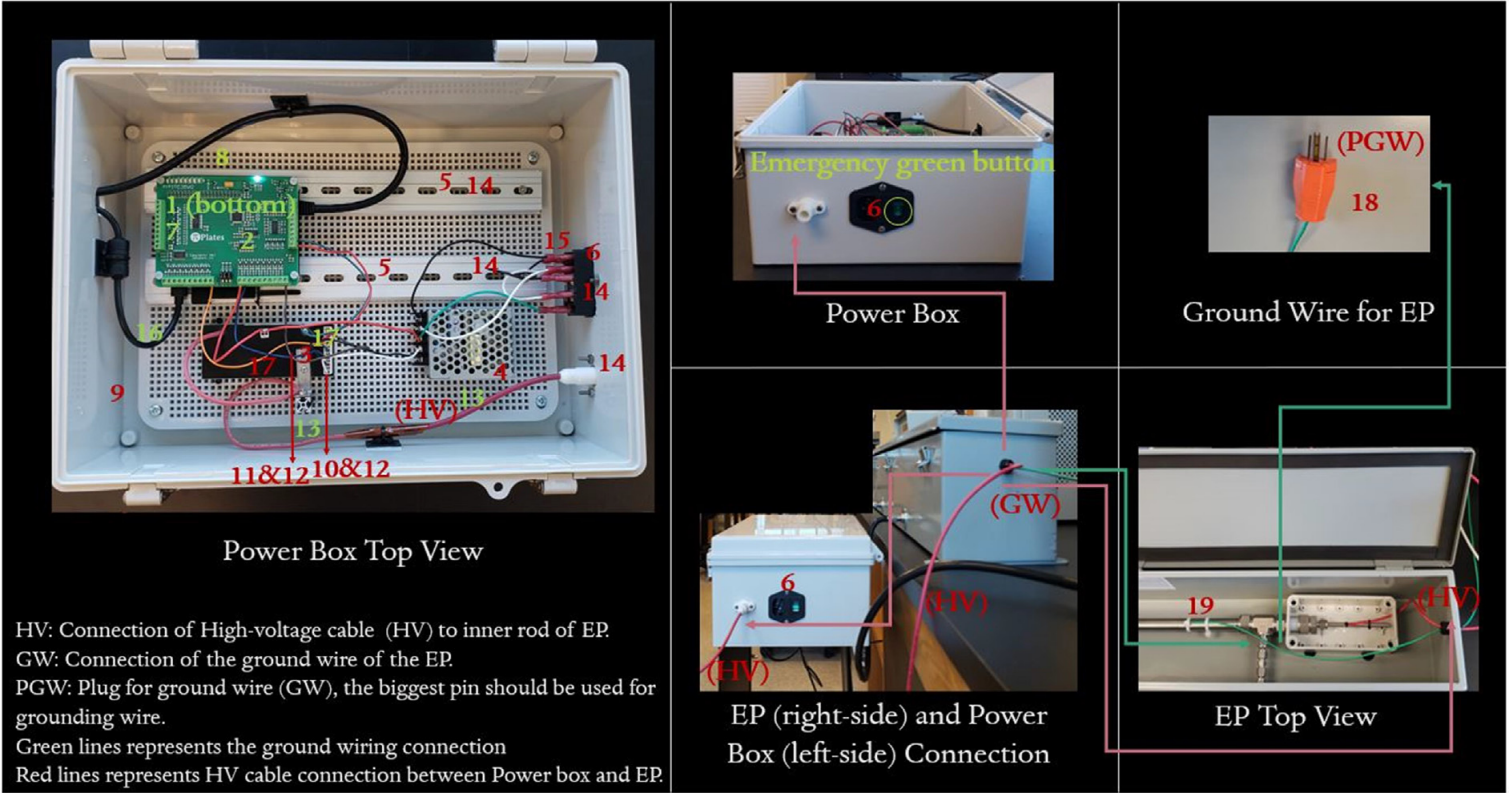
Assembled materials to operate the electronic box. The numbering is discussed in the build instructions.

The device is controlled via a custom-made high-voltage power supply box that consists of a single board computer (Raspberry Pi-4), a multifunction data acquisition board, and a variable output high-voltage DC power supply. Fig. 3 shows a schematic of the power supply box. A photograph is provided in Fig. 4. The system consists of two boxes, one for the electrostatic precipitator (Fig. 2) and a second one for the power supply box (Fig. 4). The female high voltage cable wire (HV, see in Fig. 4, Power Box Top View) in the power supply box is connected to the male high-voltage cable (middle bottom in Fig. 4) in the precipitator. The traces are given in red lines in Fig. 4. The design guidelines of the box were to minimize cost, build upon a 100% freely licensed software stack, and be versatile to allow for a re-purposing of the box for different use cases. The enclosure (14.6″ x 10.6″ x 5.9″) holds a variable 0–10 kV DC high-voltage power supply (model 10A12-P4-F-M−AT20, UltraVolt, Inc., Ronkonkoma, New York, U.S.A.), a 12 V DC power supply (Mean Well RS-15–12 Series) to power the high voltage power supply, a Raspberry Pi single-board computer (Raspberry Pi 4 Model B, Raspberry Pi Foundation, Cambridge, United Kingdom), and a DAQC2 Pi-Plate which is a multifunction data acquisition and control card (Wallyware Inc., Duluth, Georgia, U.S.A).

**Fig. 5:**
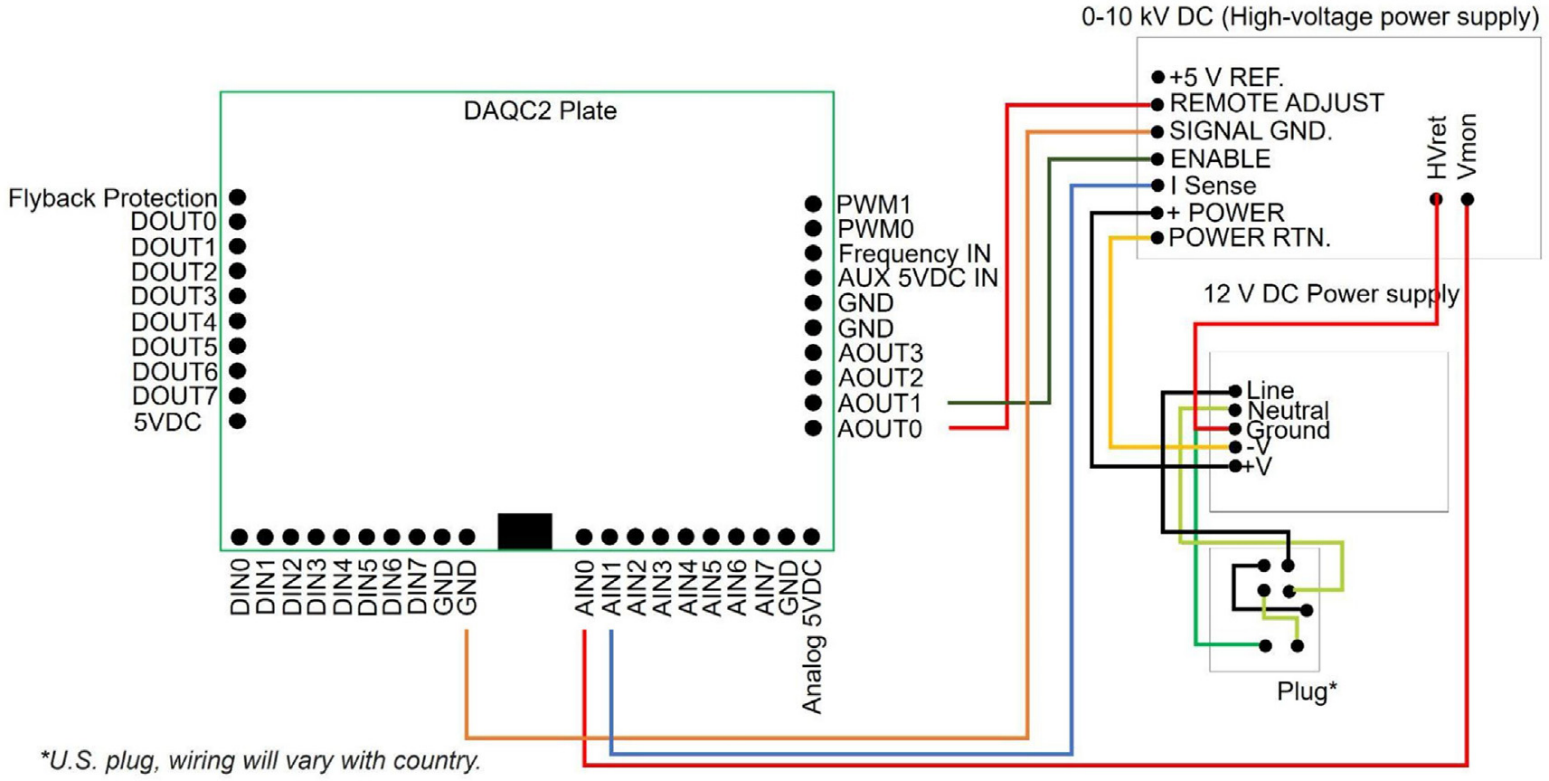
Wire diagram of the Raspberry Pi, DAQC2, and power supplies.

The DAQC2 plate provides 0 to 4.095 V 12-bit resolution digital-to-analog output, which is used to control the high voltage output from the UltraVolt power supply between 0 and 8825 V. The high voltage power supply output voltage and output current are monitored using 16-bit resolution analog-to-digital inputs. The wiring diagram of the DAQC2 plate is provided in Fig. 5. The selected Raspberry Pi operating system image is the 64-bit version of Raspberry Pi OS (derived from Debian Linux), running on a 64 GB microSDXC UHS-I memory card. Software written in the Julia language provides a graphical user interface that is used to set the voltage, displays a time series of voltage and current readings, and saves the data to disk. The graphical user interface can be accessed remotely from any computer via a secure shell connection and a locally installed X-server.

• The novelty of this work is that the design is fully open (See Table 1). Only widely available off-the-shelf products are used. Assembly requires only standard tools. One design criterion was to reduce the cost of the device. In literature, lowering the cost of a device (e.g. sensor) would provide the detection of the source contribution of the aerosols [24], and would fill the gaps between the economical deficiency and science [25]. Opportunities for additional cost savings are discussed at the end of this work.
• Another design consideration was to keep the components modular and re-usable so that the materials can be repurposed for other uses after the project is completed. The device is simple enough that a graduate student without an engineering background can build it as part of a research project.
• Open designs such as the one presented here ensure that the science is accessible and that the instrumentation is affordable to a wider audience. It also provides an opportunity for the design to be improved upon [26], [27].
• The cost associated with building this device is less than that of the commercially available analog.

## Design files summary

**Table 1:** Design file list including technical drawing of the EP, assembled parts of EP and electronic box, connectors, software screenshot, wire diagram, and software source code.

Design file name	File type	Open source license	Location of the file
EP_1technical_drawing	pptx	CC BY-NC-SA 4.0 International	https://doi.org/10.5281/zenodo.5295817
EP_2assembled	pptx	CC BY-NC-SA 4.0 International	https://doi.org/10.5281/zenodo.5295817
EP_3schematic_high_voltage_power_supply_box	pptx	CC BY-NC-SA 4.0 International	https://doi.org/10.5281/zenodo.5295817
EP_4assembled_high_voltage_power_supply_box	pptx	CC BY-NC-SA 4.0 International	https://doi.org/10.5281/zenodo.5295817
EP_5wire_diagram	pptx	CC BY-NC-SA 4.0 International	https://doi.org/10.5281/zenodo.5295817
zapperDAQ	multiple	GPL-3.0 License	https://doi.org/10.5281/zenodo.5295725
DAQC2Plate	multiple	GPL-3.0 License	https://doi.org/10.5281/zenodo.5295711
openHardwareDesignPrecipitator2021	multiple	GPL-3.0 License	https://doi.org/10.5281/zenodo.5295817

EP_1technical_drawing.pptx corresponds to Fig. 1.

EP_2assembled.pptx corresponds to Fig. 2.

EP_3schematic_high_voltage_power_supply_box.pptx corresponds to Fig. 3.

EP_4assembled_high_voltage_power_supply_box.pptx corresponds to Fig. 4

EP_5wire_diagram: EP_5wire_diagram.pptx corresponds to Fig. 5.

zapperDAQ: The data acquisition and control software repository.

DAQC2Plate: Julia wrapper of the data acquisition plate device driver.

openHardwareDesignPrecipitator2021: Repository includes data files and processing scripts.

## Bill of materials summary

Bill of materials to construct the EP, including the electronic box, and cabling connections are provided in Table 2, Table 3, and Table 4, respectively. The items used in the bill of materials are not unique and substitutions for already available parts or from different vendors are possible. Small changes in dimensions will not affect the basic performance of the device.

**Table 2:** Bill of materials to construct the EP.

Designator	Component	Number	Cost per unit - currency(USD)	Total cost –currency (USD)	Source of materials	Material type
McMaster-Carr 89785K262	Smooth-Bore Seamless 316 Stainless Steel Tubing, 3/4″ Tube OD, 0.065″ Wall Thickness, 1′ Long	1	15	15	McMaster-Carr	Stainless Steel
McMaster-Carr 8936K133	Tight-Tolerance Corrosion-Resistant 316/316L Stainless Steel, 1/4″ Diameter, 3′ Long	1	19	19	McMaster-Carr	Stainless Steel
McMaster-Carr 1988T11	Slippery UHMW Polyethylene Tube, Tight-Tolerance, 3/4″ OD, 1/4″ ID, 6″ Long	1	16	16	McMaster-Carr	Polyethylene
McMaster-Carr 7301K13	Electronics Corrosion-Resistant Washdown Enclosures	2	17	34	McMaster-Carr	ABS plastic
Swagelok SS-1210-3-12-6	Stainless Steel Swagelok Tube Fitting, Reducing Union Tee, 3/4 in. × 3/4 in. × 3/8 in. Tube OD	2	82	164	Swagelok	Stainless Steel
Swagelok SS-1210-6-4BT	Stainless Steel Swagelok Tube Fitting, Bored-Through Reducing Union, 3/4 in. × 1/4 in. Tube OD	2	20	40	Swagelok	Stainless Steel
Swagelok T-1213-1	PTFE Front Ferrule for 3/4 in. Swagelok Tube Fitting	6	5	30	Swagelok	Polytetrafluoroethylene (PTFE)
Swagelok T-1214-1	PTFE Back Ferrule for 3/4 in. Swagelok Tube Fitting	6	5	30	Swagelok	Polytetrafluoroethylene (PTFE)
Swagelok NY-400-SET	Nylon Ferrule Set (1 Front Ferrule/1 Back Ferrule) for 1/4 in. Swagelok Tube Fitting, Please order in multiples of ten	8	1	8	Swagelok	Polytetrafluoroethylene (PTFE)
Swagelok SS-400-6	Stainless Steel Swagelok Tube Fitting, Union, 1/4 in. Tube OD	2	12	24	Swagelok	Stainless Steel
Swagelok SS-400-61	Stainless Steel Swagelok Tube Fitting, Bulkhead Union, 1/4 in. Tube OD	2	19	38	Swagelok	Stainless Steel
Swagelok SS-401-PC	Stainless Steel Swagelok Tube Fitting, Port Connector, 1/4 in. Tube OD	4	6	24	Swagelok	Stainless Steel
Hammond Manufacturing 1486D36	HAMMOND 1486 SERIES WIRING TROUGH - NEMA 12	1	182	182	Hammond Manufacturing	14 gauge steel
Blue Hawk 604016	Blue Hawk 25-Count 1/2-in × 1-1/2-in Zinc-Plated Standard (SAE) Fender Washers	4	7	6.98	Blue Hawk	Metal
Swagelok SS-601-PC-4	Stainless Steel Swagelok Tube Fitting, Reducing Port Connector, 3/8 in. × 1/4 in. Tube OD	2	9	18	Swagelok	Stainless Steel
Swagelok SS-402-1	316 Stainless Steel Nut for 1/4 in. Swagelok Tube Fitting	4	2	8	Swagelok	Stainless Steel

**Table 3:** Bill of materials to construct the power supply.

**Designator**	**Component**	**Number**	**Cost per unit - currency(USD)**	**Total cost – currency (USD)**	**Source of materials**	**Material type**
Gibot JX0039	GiBot Cable Glands - 25 Pack Plastic Waterproof 3.5-13mm Cable Glands Joints Wire Protectors, PG 7/9/11/13.5/16, Black	25pack	10	10	Gibot	Nylon PA6
Raspberry Pi Foundation Model 4 b	Raspberry Pi 4	1	65	65	Mouser	Multiple
Wallyware Inc. DAQC2plate	Data Acquisition and Controller DAQC2plate	1	50	50	Wallyware Inc.	Multiple
Ultravolt 10A12-P4-F-M-AT20	10kV High Voltage Biasing Supply	1	584	584	Digikey	Multiple
Mean Well RS-15-12	DC Power Supply	1	9	9	TRC electronics	Multiple
T&G D357A11-305(2)	35 mm DIN Rail	2-rail	9	9	T&G	Aluminum
Twidec IEC320GG	Twidec/2Pcs 10A 250V 3 Pin Inlet Module Plug 5A Fuse Switch Male Power Socket With Green Boat Rocker Switch IEC320GG	2-pack	10	10	Twidec	Plastic
SanDisk SDSQUB3-064G-ANCIA	Ultra PLUS 64GB microSDXC UHS-I Memory Card	1	15	15	SanDisk	Other
-	Ethernet cable	-				Other
Qilipsu QL-372715AGH	QILIPSU Hinged Cover Stainless Steel Latch 370x270x150mm Junction Box with Mounting Plate, ABS Plastic DIY Electrical Project Case IP67 Waterproof Dustproof Enclosure Grey (14.6“x10.6”x5.9“)	1	58	58	Qilipsu	Multiple
Molex 10112133	CONN HOUSING 13POS .100 HI PRESS	1	1	1	Digikey	Multiple
Molex 10112023	Headers & Wire Housings HOUSING 2 POS	1	1	1	Mouser	Plastic
Molex 08-55-0124	Headers, SOCKET 22-30AWG REEL Reel of 5000	100-pack	1	1	Allied electronics	Brass
-	Screws	-	-	-	-	Steel
-	Screw/washer	-	-	-	-	
Panduit DNF18-250FI-M	Terminals FREC-DSC 22-18 250X032 NYL RED	1-pack	5	5	Digikey	Multiple
Raspberry Pi Foundation KSA-15E-051300HU Black	Raspberry Pi 15.3W USB-C Power Supply	1	8	8	Digikey	Multiple
BNTECHGO FBA_SW20G10008F05C7	Cable wires, 20 AWG	1-pack	10	10	BNTECHGO	Multiple
LevitonR51-515PV-0OR	Ground plug, 15 Amp 125-Volt 3-Wire Plug, Orange	1	3	3	Leviton	Multiple
Southwire E51583 - AWG 14	Ground wire, Southwire E51583 F 90deg Cwire AWG 14	1	60	60	Southwire	Multiple

**Table 4:** Bill of materials to construct the connecting cabling.

Designator	Component	Number	Cost per unit - currency(USD)	Total cost – currency (USD)	Source of materials	Material type
-	Ethernet cable	-	-	-	-	Other
Molex 10112133	CONN HOUSING 13POS .100 HI PRESS	1	1	1	Digikey	Multiple
Molex 10112023	Headers & Wire Housings HOUSING 2 POS	1	1	1	Mouser	Plastic
Molex 08-55-0124	Headers, SOCKET 22-30AWG REEL Reel of 5000	100-pack	1	1	Allied electronics	Multiple
-	Screws	-	-	-	-	Steel
-	Screw/washer	-	-	-	-	-
Panduit DNF18-250FI-M	Terminals FREC-DSC 22-18 250X032 NYL RED	1-pack	5	5	Digikey	Multiple
Raspberry Pi Foundation KSA-15E-051300HU Black	Raspberry Pi 15.3W USB-C Power Supply	1	8	8	Digikey	Multiple
BNTECHGO FBA_SW20G10008F05C7	Cable wires, 20 AWG	1-pack	10	10	BNTECHGO	Multiple
LevitonR51-515PV-0OR	Ground plug, 15 Amp 125-Volt 3-Wire Plug, Orange	1	3	3	Leviton	Multiple
Southwire E51583 - AWG 14	Ground wire, Southwire E51583 F 90deg Cwire AWG 14	1	60	60	Southwire	Multiple

## Build instructions

Fig. 2 shows a photograph of the fully assembled EP. The numbers indicated in instructions correspond to the numbers in Fig. 2. Required tools include wrenches covering the sizes 1/4″, 9/16″, and 3/4″ or two adjustable wrenches, a drill, a metal tube cutter, and screwdrivers. Personal protective equipment should be worn when working with power tools, including goggles and gloves.

1 Cut the polyethylene tube (#3) in half to get two polyethylene tubes 3″ long.
2 Cut the stainless steel rod (#2) with a metal tube cutter to the desired length. We used 29″. Avoid creating any deformation on the rod during the cutting process.
3 Place the stainless steel rod (#2) into the outer 3/4″ tube (#1) to create two concentric cylinders.
4 Insert the concentric cylinders (#1 and #2) into one side of the 3/4″ x 3/4″ x 3/8″ union tee fitting (#5) having placed back and front nylon ferrules of 3/4″ size (#7 and #8).
5 Tighten the nut with wrenches 1/8 turn past finger tight. Insert the polyethylene tube (#3) into the other side of the 3/4″ x 3/4″ x 3/8″ union tee fitting (#5) until the front ferrule seats against the body of the 3/4″ nuts.
6 Tighten the nut with wrenches 1/8 turn past finger tight.
7 Drill a hole into the plastic enclosure (#4).
8 Pass the polyethylene tube through the drilled hole and insert polyethylene tube with 3/4″ back and front nylon ferrules (#7 and #8) until the front ferrule seats against the body of the 3/4″ nuts of the reducing union of 3/4″ x 1/4″ (#6). This step should be inside of the plastic enclosure (#4).
9 Repeat the previous step for the other side of the cylinders too.
10 Pass the 1/4″ back and front nylon ferrules (#9) through the rod between the 1/4″ nut (#16) and reducing union (#6). Make sure to tighten all the connections.
11 Drill two holes into the metal enclosure box (#13) for both inlet and exit of the flows.
12 Place the reducing port connector (#15) between the 3/8″ nuts and 1/4″ nuts.
13 Connect the 1/4″ union (#10) to the 1/4″ nut (#16) with a bulkhead union (#11) which should pass through the drilled hole.
14 Tighten all the connections 1/8 turn past finger tight using wrenches.
15 Use washers (#14) for the drilled holes for both sides of the bulkhead union (#11) then tighten the union connections.
16 Place a 1/4″ nut for the inlet of the EP by using a port connector (#12).
17 Repeat the steps for the outlet side of the flow.

Fig. 4 shows a photograph of the fully assembled power supply box. The numbers indicated in instructions correspond to the numbers in Fig. 4. A screwdriver, drill, wire terminal and crimper, and wire stripper are necessary tools for assembly. A voltmeter is needed for testing. To start the assembly. Instructions for MOLEX connections are given below;

1 The connection between the power supply (#3) and Raspberry Pi is done using MOLEX connectors which include rectangular connectors (housings) (#10 and #11), headers (#12), and colored wires (#17).
2 For the MOLEX connectors, the user needs to have a MOLEX-type wire cutter and a MOLEX-type hand crimper (for 10-22AWG according to the wire selection).
3 First, match the wire gauge with a labeled hole on the wire cutter then strip the wire with a wire cutter by placing the wire into the proper hole. Cut the insulation of the wire. Slide the naked wire into the header (#12).
4 Use the compression tool side of the crimper to slide the wire with the connector until they sit and compress the crimper to lock the header to the wire.
5 Put slightly the locked wire connection into the housing (#10 or #11).
6 Then repeat the previous steps for all colored wires (#17) to complete the power supply (#3) connection.
7 Drill a hole into the ABS plastic enclosure box (#9) for the power switch button (#6) and outlet of the electrical socket for the EP (see further discussion below). Female nylon fully insulated connectors should be used for the wiring connection of the power switch button (#6). (The power switch needs to be ordered and wired to match the country's power grid).
8 Then, make proper holes into the mounting plate (a part of #9), for the DIN rails (#5), and power supplies (#3 and #4).
9 All wire connections are recommended to be completed outside the box before the last installation.
10 Connect ground wire (#19) to the ground pin on the electrical plug (the biggest pin of the orange plug, see #18 in Fig. 4), and make sure to connect the ground pin to the ground wire of the plug.
11 Use zip ties to connect the ground wire to the outside of the EP.
12 Use a zip tie to connect a high-voltage cable (see further below, (HV) in Fig. 4 HV) to the inner rod. Attach the DIN rails (#5), Raspberry Pi (#2), DAQC2plate (#1), and power supplies (#3 and #4) to the bottom of the box.
13 Connect the signal wires according to the wiring diagram given in Fig. 5.
14 The wire installation step can be completed after placing the mounting plate into the box.
15 After the last installation, it is crucial to test the continuity of the system to ensure that it is grounded and not shorted before powering up the device.

**Fig. A1:**
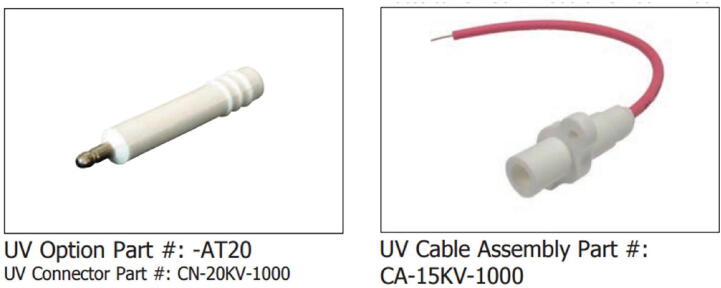
Connectors used in our system.

The Ultravolt power supply is delivered with a custom connector specified in the order. We recommend selecting the desired connector and cable length when ordering the power supply. The “AT20” in the Ultravolt 10A12-P4-F-M-AT20 refers to the connector on the power supply output, which mates to the CA-15KV-1000. These options are shown in Fig. A1. The power supply was originally ordered to interface with a different instrument. However, for maximum safety, it is more desirable to have the CA-15KV-1000 connector facing outward from the box (14 in Fig. 4). We, therefore, cut the UV connector and spliced the high-voltage output from the power supply with the CA-15KV-1000 assembly. The splice-point is insulated with regular electrical tape and several layers of Kapton tape (Polyimide). A second high voltage cable connects the EP to the box. We had obtained a number of these cables from a surplus sale and thus had the cable on hand. The connector corresponds to the Amphenol-Alden A400QX“L” series. The connector/cable assembly can be ordered directly from Amphenol-Alden, independently of the power supply. Although the cable assembly is relatively inexpensive (∼$40), the company requires a minimum order of $750 for custom cables. We, therefore, recommend either request all extra cables when ordering the power supply or discuss other options with the company's sales representative*.*

## Operation instructions

After the installation, the safety issue is important for the operation of EP. Before plugging in (Fig. 4 #18) and switching on EP (Fig. 4 #6), make sure to pursue the following steps of 1) software installation and 2) high-voltage safety.

### Software

**Fig. 6:**
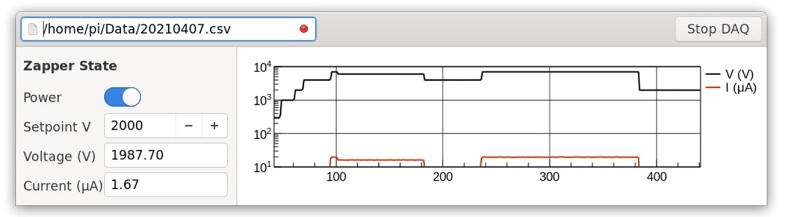
Screenshot of the graphical user interface.

The data acquisition and control software is stored on GitHub https://github.com/mdpetters/zapperDAQ. The README.md file includes detailed installation instructions. Fig. 6 shows a screenshot of the user interface. The user interface allows the user to toggle the power using an on/off switch and set the voltage. The on/off switch sends a voltage to the “ENABLE” pin (Fig. 5) and provides a software means to power down the device. The setpoint voltage sends the appropriately scaled voltage to the “REMOTE ADJUST” (Fig. 5). The system reads the reported high voltage and current from the respective pins. The time series displays the measured current and voltage. Note that the read voltage by the DAQC2Plate is biased. The reasons are not fully clear, but might be related to an impedance mismatch. Calibration is used in the software to correct for the bias. We recommend performing your own calibration between the setpoint voltage and the readout. We have observed no bias when reading the HVmon pin with a standard voltmeter or with a different multifunction DAQ device, such as the LabJack U3 or LabJackU6. Performing a precise voltage calibration is critical when generating low voltages (<200 V) and when voltage accuracy is important for the application (e.g when using the system to power a scanning mobility particle sizer). The calibration is less critical when the objective is limited to removing all particles. The primary purpose for displaying the readout voltage is to provide user feedback on the instrument state. Arcing will result in unstable readout voltage. Corona effects will show an unsteady readout current.

The current monitor is directly read by the analog-to-digital converter. Per specification of the power supply model, the current monitor includes a leakage current from the internal resistors. We do not subtract the leakage current in the software and thus the displayed current scales with the applied voltage. In the case of corona formation, the current visibly increases and the time series becomes noisy. For applications that require precise characterization of the current, users can subtract the leakage current in software, build an analog summing circuit, or invest in the “-I5 interface option” which provides improved monitoring capabilities.

### High-Voltage safety

The safety advice here is provided for information. Users are responsible for conducting their own risk assessment. Users operate the device at their own risk. It is the sole responsibility of the user to follow all federal, state, local, and institutional safety protocols. We strongly recommend a safety audit by appropriately trained personnel before powering up the device for the first time.

• Electrical hazards may cause several damages to the human body. Electrical hazards include all power connections from the AC outlet and the high voltage applied to the electrostatic precipitator. Follow the standard safety protocols when working with 110 V/220 V AC power.
• The electrostatic precipitator's highest normal operating voltage is 3 kV DC, which refers to a high voltage platform during handling. However, the power supply may produce up to 10 kV output. For this reason, all calculations are carried to 10 kV. To reduce the electric shock of the system the high voltage supplies, the electrical cables, and Raspberry Pi are enclosed in an acrylonitrile butadiene styrene (ABS) plastic box. Parts with exposed high voltage are placed inside a secondary ABS enclosure. ABS plastic has a dielectric strength between 16.7 kV/mm [28]. The ABS plastic boxes have a thickness of at least>1 mm and are expected to insulate the user from electrical hazards even in the case of catastrophic failure (e.g. a detached wire).

According to the most current international standard for Safety requirements for electrical equipment for measurement, control, and laboratory use BS EN 61010–1:2010 + A1:2019 [29], the DC voltages present in dry locations exceeding 70 Volts up to 15 kV is considered hazardous only if the current exceeds 2 mA DC or if the stored charge exceeds 45 μC[29]. In our high-voltage supply (#3 in Fig. 4), the highest voltage is 10 kV, and the current is 0.4 mA (for the Entire Output Voltage Range specified in the online datasheet). For additional safety requirements, a similar limiting consideration is applied to the EP where the stored charge on the rod is also calculated for the highest voltage value of 10 kV similar to the study of McGinness et al. (2019)[30]. The stored charge (Q in Coulombs) of the inside rod of the EP is Q = CxV where V is the voltage (in Volt) and C is the capacitance (in Farad). The capacitance is C = $\frac{2\pi ∊L}{ln\left(\frac{b}{a}\right)}$where ∈ is the permittivity of air 8.85x10^−12^F/m between the inner and outer rode, L = 0.3048 m (12″, the length of the cylinder), b = 0.007874 m (outer electrode radius), and a = 0.003175 m (rod radius). So the capacitance of the rod is 18.7 pF and the stored charge on the rod for 10 kV would be 18.7 pF × 10000 V = 0.187 μC. Thus, the calculated stored charge of the rod of EP with a high-voltage supply for 10 kV is also well below the standard values.

Please note that the user should frequently inspect all cable connections. Always switch off all the power supplies and detach the main power cable before servicing the instrument. Even if the current is low, never touch cables, connectors, or power supplies during operation. Disconnect the plug or switch off the green button in case of emergency (see Fig. 4, #6). To avoid any injuries, it is important to always keep the cables and high voltage supplies dry.

The EP box is grounded together with the outer rod. The polyethylene tube isolates the device on both sides. The edge points of the rods are also secured with ABS plastic boxes to provide further safety. Never open the secondary enclosures or touch the inner rod while in operation. At an operating voltage > 3 kV the system may arc. The excess current is deposited through the ground. To avoid unwanted accidental arcs, we strongly recommend testing the control voltage sent by the software with a voltmeter to ensure that the signal lines are working correctly.

## Validation and characterization

### Theory of the EP

Electrical mobility (*z*) can be simply defined by the movement of an electrically charged particle in an electric field [31]. In other words, electrical mobility is the ratio of the terminal velocity of the particle (the velocity calculated by the balance between the drag force and the electrostatic force) and the electrical field [31].

The relationship between mobility and mobility diameter is:

(1) $$z=keC_{c}\left(D\right)/3\pi \eta D=keC_{c}\left(D_{\mathrm{ve}}\right)/3\pi \eta \chi D_{\mathrm{ve}}$$

where *z* is the electrical mobility, *k* is the number of charges on the particle, *e* is the elementary charge, *C*_c_ is the Cunningham slip flow correction factor, *η* is the fluid viscosity, χ is the dynamic shape factor which is used due to increases drag forces for non-spherical cases, and *D* is the mobility diameter. *D*_ve_ is the volume equivalent diameter and for non-spherical particles it is considered as a diameter that particle would have if it were melted to form a droplet including the voids in its shape [32]. For spherical particles χ = 1 and the mobility diameter and particle diameter are identical. Non-spherical particles have well-defined mobility diameter, and χ > 1 [23]. Typical values for χ are1.08 for cubical particles and up to 1.88 for agglomerates (e.g talc) [31].

The DMA is used to size select particles based on their electrical mobility. As a first approximation, the tube can be modeled as a rudimentary version of a cylindrical DMA. For the cylindrical DMA and balanced flows, where the inlet and outlet flows have an equal flow rate throughout the instrument, the particle mobility selected by a DMA is given by [33]:

(2) $$z^{s}={(q}_{\mathrm{sh}}/2\pi LV)*ln\left(r_{2}/r_{1}\right)$$

where *z*^s^ (m^2^/Vs) is the selected mobility, *q*_sh_ is the flow rate, *L* is the column length, *r*_1_ and *r*_2_ are the radii of the inner and outer section of the annulus, and V is the potential applied between the two electrodes. Under given physical dimensions (constant L and r), particle electrical mobility becomes a function of *q*_sh_ and V. Larger z^s^ corresponds to a smaller mobility diameter. The precipitator does not have a sample flow or separate exit slit. As will be shown later using numerical simulation, Equation (2) predicts the critical mobility for the EP. Particles with electrical mobility larger than *z*^s^ will all be removed. Some fraction of particles with electrical mobility less than *z*^s^ will transmit. The largest completely removed mobility diameter is computed by applying *z*^s^ to Equation (1). We define the transfer function such that the function defines the probability of a charged particle to leave the instrument along the stream at applied voltage [33]. For the electrostatic precipitator, we expect the transfer function to scale with the ratio *z*/*z*^s^.

### Trajectory model

The particle trajectory through a cylindrical capacitor is given by a set of coupled differential equations [34], [35].

(3) $$\frac{\mathrm{dr}}{\mathrm{dt}}=zE\left(r,V\right)$$

(4) $$\frac{\mathrm{dx}}{\mathrm{dt}}=u_{x}\left(r\right)$$

where $E\left(r,V\right)=\frac{V}{\mathrm{rln}r_{2}/r_{1}}$ the radial electric field at position *r*, and *u*_x_(r) is the streamwise velocity at position *r*. The velocity profile *u*_x_(r) in the annulus is parabolic. The analytical steady-state solution for an incompressible, laminar flow of a Newtonian fluid in an infinitely long pipe annulus is given in the supplement of ref. [35]. Equations (3), (4) can be integrated numerically to find the particle trajectory through an idealized precipitator, assuming steady-state flow and neglecting entrance effects.

We modeled the trajectory of particles through the EP precipitator with dimensions *r*_1_ = 1/8″ (inner electrode radius), *r*_2_ = 3/8″ (outer electrode radius), and *L* = 12″ (nominal electrode length). Particles of two different mobilities (colors) are released at three different starting locations. The assumed flow rate and voltage are 1 L min^−1^ and 346 V, respectively. The mobility of blue trajectories corresponds to *z*^s^ and is computed using Eq. (1). The mobility of the yellow trajectory is 1/2 the value of the blue trajectory.

**Fig. 7:**
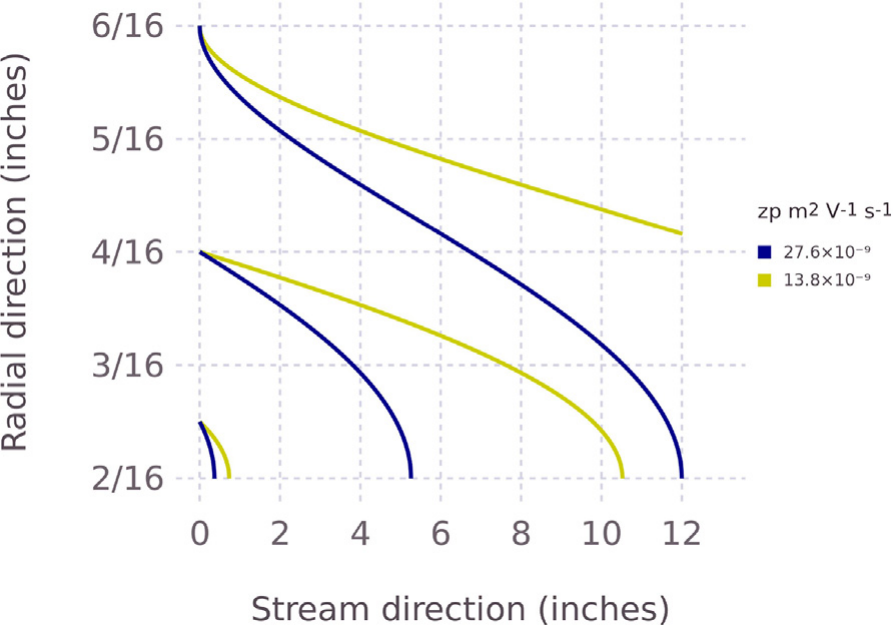
Modeled trajectory of particles through the EP precipitator with dimensions *r*_1_ = 1/8″ (inner electrode radius), *r*_2_ = 3/8″ (outer electrode radius), and *L* = 12″ (nominal electrode length). Particles of two different mobilities (colors) are released at three different starting locations. The assumed flow rate and voltage is 1 L min^−1^ and 346 V, respectively. The mobility of blue trajectories corresponds to *z*^s^ and is computed using Eq. (1). The mobility of the yellow trajectory is 1/2 the value of the blue trajectory. The corresponding particle sizes for blue and yellow lines are 100 nm and 150 nm, respectively. (For interpretation of the references to color in this figure legend, the reader is referred to the web version of this article.)

The mobility parameter accounts for the electrical field strength, sheath flow, and physical dimensions of the precipitator for the ideal case. Thus, a particle’s radial position at the entrance of the precipitator will be the determining factor on whether it is removed from the flow. Unlike a DMA, the EP has no particle-free sheath flow and no exit slit. Without the sheath flow, particles are present at all radial coordinates, presumably at a uniform distribution. Fig. 7 shows example simulations for particles released at the three radial positions at the inlet of the instrument. Particles released closer to the center electrode are all captured by the wall of the inner electrode. The particles having mobility *z*^s^ starting the furthest from the center electrode impact on wall of the inner electrode just before the exit of the column. Particles having mobility *z* < *z*^s^ starting the furthest from the center electrode do not reach the wall of the electrode and will transmit. However, particles with the same mobility released closer to the center rod will be captured. Hence a fraction of particles having *z* < *z*^s^ will transmit. Repeating the calculation for 200 trajectories released between *r*_1_ < *r* < *r*_2_ and evaluating the weighted fraction of trajectories not reaching the wall of the electrode is a measure of the expected transmission efficiency. The weight accounts for the fraction of fluid passing through the pipe for a given streamline.

**Fig. 8:**
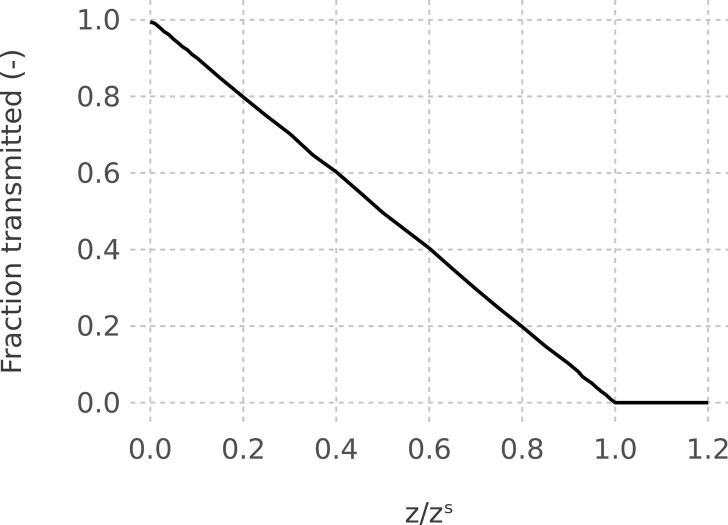
Ideal transfer function of the cylindrical EP.

The ideal transfer function of the cylindrical EP Fig. 8 shows the transfer function for the ideal cylindrical EP. Zero transmission is observed for *z* > *z*^s^. Transmission linearly increases with decreasing *z*/*z*^s^. In Fig. 7, particles are attracted to the inner rod. Reversing the polarity will deposit the same particles on the outer cylinder. The modeled ideal transfer function shown in Fig. 7 is independent of the direction of the electrical drift velocity, i.e. of the polarity of the power supply. As will be shown later, the ideal transfer function is not observed. Computational fluid dynamics (CFD) simulations are used to assess differences between the ideal precipitator and design used here.

### Computational fluid dynamics (CFD) simulations

Significant entrance and exit distortions of the transfer profile are expected due to the placement of the inlet perpendicular to the streamwise flow. Simulations of the 3D flow profile were carried out using the OpenFOAM v8 free-software toolbox. The annulus gap was discretized into an algorithmically created structured cylindrical grid with 151,334 hexahedral cells. Flow velocity at the inlet was set such that the average volumetric flow rate through the gap was 1 L min^−1^. The outlet pressure is fixed. Flow velocity at the walls was set to zero. Initial flow velocity inside the domain volume was set to zero. Simulations were carried out using the icoFoam solver, which applies the PISO algorithm [36] to solve for the evolution of the incompressible flow through the domain. Simulations were carried out for 4 s. Fluid trajectories were computed using the Runge-Kutta method. The simulation case files (mesh generation and solver settings) are easily adaptable to simulate altered geometries and are made available with the data repository.

**Fig. 9:**
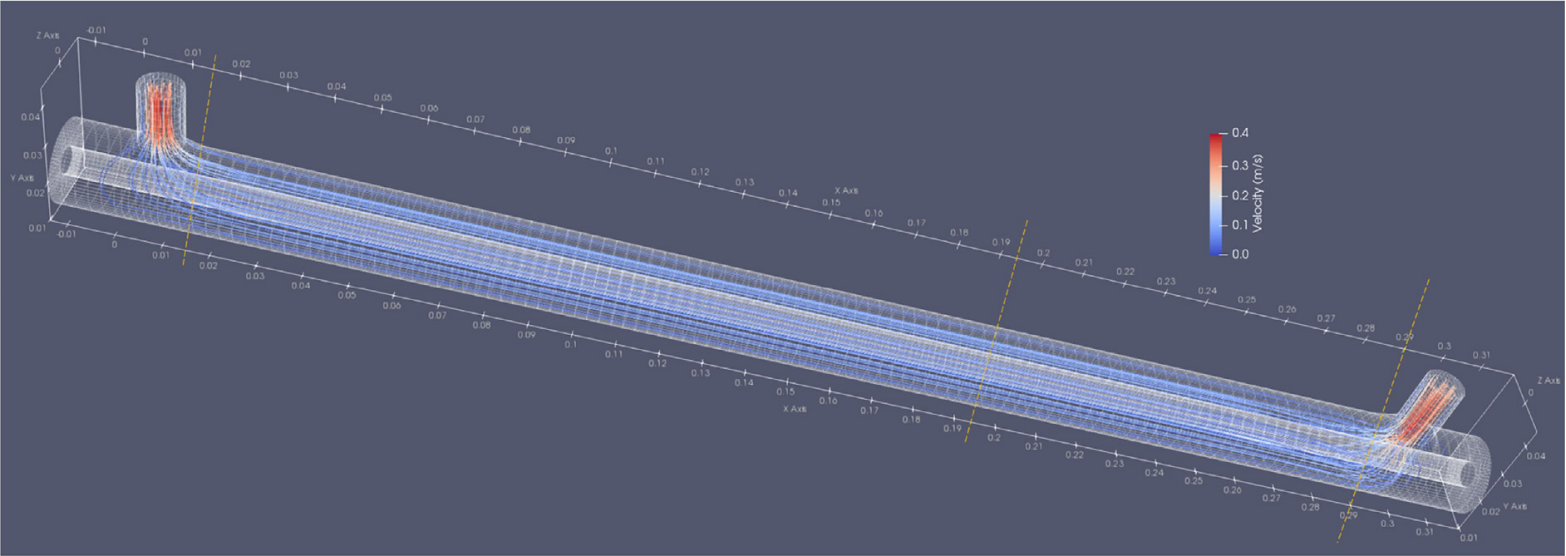
Fluid dynamic simulation domain and fluid streamlines for a precipitator with dimensions *r*_1_ = 2/16″ (inner electrode radius), *r*_2_ = 3/8″ (outer electrode radius), *L* = 12″ (nominal electrode length), *r*_i_ = 3/16″ (inlet radius). X, Y, and Z axes are shown in [m].

Fig. 9 shows the model domain for the CFD simulations. The inlet (left) and outlet (right) are modeled to represent the Swagelok T-fitting. At the entrance to the tube, the flow decelerates. Streamlines wrap around the inner cylinder to make up the flow along the tube on the side opposite the inlet. At ∼ 3 cm past the inlet, the 3D flow profile is fully equilibrated. Similarly, ∼3 cm before the outlet the flow accelerates and the streamlines curve toward the outlet of the device. Some of the streamlines flow into the dead volume to the left of the inlet and to the right of the outlet.

**Fig. 10:**
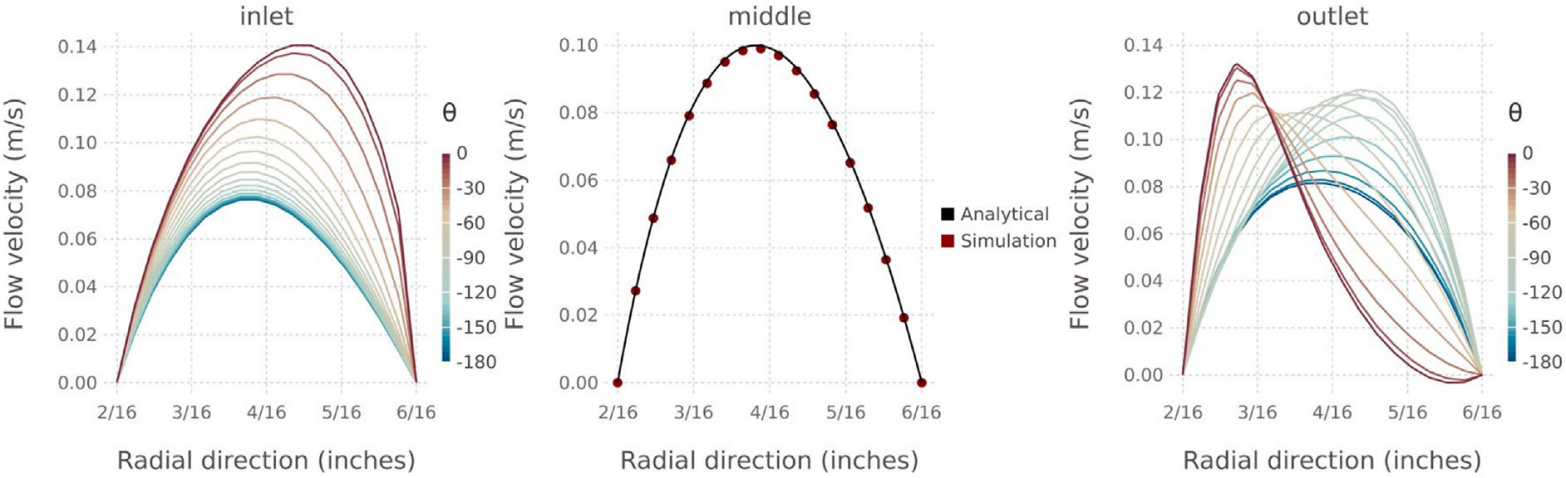
Streamwise velocity as a function of the radial coordinate near the inlet (0.015 m), the middle (0.194 m), and near the outlet (0.29 m) of the domain as predicted by the CFD simulation. The color scale for the inlet/outlet panels corresponds to the angular coordinate in a cylindrical coordinate system with (*x*,*r*,*θ*), where × is as in Fig. 7, *r* is the radial distance from the centerline, and *θ* is the angle. *θ* = 0° aligns with the z-axis in Fig. 7 and corresponds to the inlet side of the precipitator. *θ* = -180° aligns with the backside of the precipitator. Color in the middle panel corresponds to the analytical solution for the incompressible, laminar flow of a Newtonian fluid in an infinitely long pipe annulus (black) and the velocity at cell interfaces (red points). (For interpretation of the references to color in this figure legend, the reader is referred to the web version of this article.)

Fig. 10 shows the radial velocity distribution at three x-coordinates within the domain: close to the entrance and exit of the system and in the center where the flow is laminar. Near the entrance and exit, the fluid velocity is maximized at the side facing the inlet/outlet (see yellow dashed lines in Fig. 9 for reference). Flow velocity at the backside of the precipitator is reduced. In the center section of the domain, the modeled velocity distribution is within 1% of the analytical solution for the incompressible, laminar flow of a Newtonian fluid in an infinitely long pipe annulus.

The CFD simulations highlight several important points. Flow distortion near the entrance and exit is significant. Residence time and the electric field strength depend on the fluid trajectory. Since the inner electrode carries charge all the way through, and since the electric field near the inlet is distorted, the effective length of the precipitator will depend on the inlet geometry and to some extent on the flow rate. The dependence on flow rate is because the entrance length depends on the Reynolds number, which is set by the inlet diameter and flow rate. Some particles will be trapped in the volume to the left of the inlet and to the right of the outlet. Since the electric field remains active there, particles with long residence times in those areas will be lost. From the CFD simulation, it is expected that the true transmission efficiency is less than the ideal transmission efficiency shown in Fig. 8.

### Experimental method

**Fig. 11:**
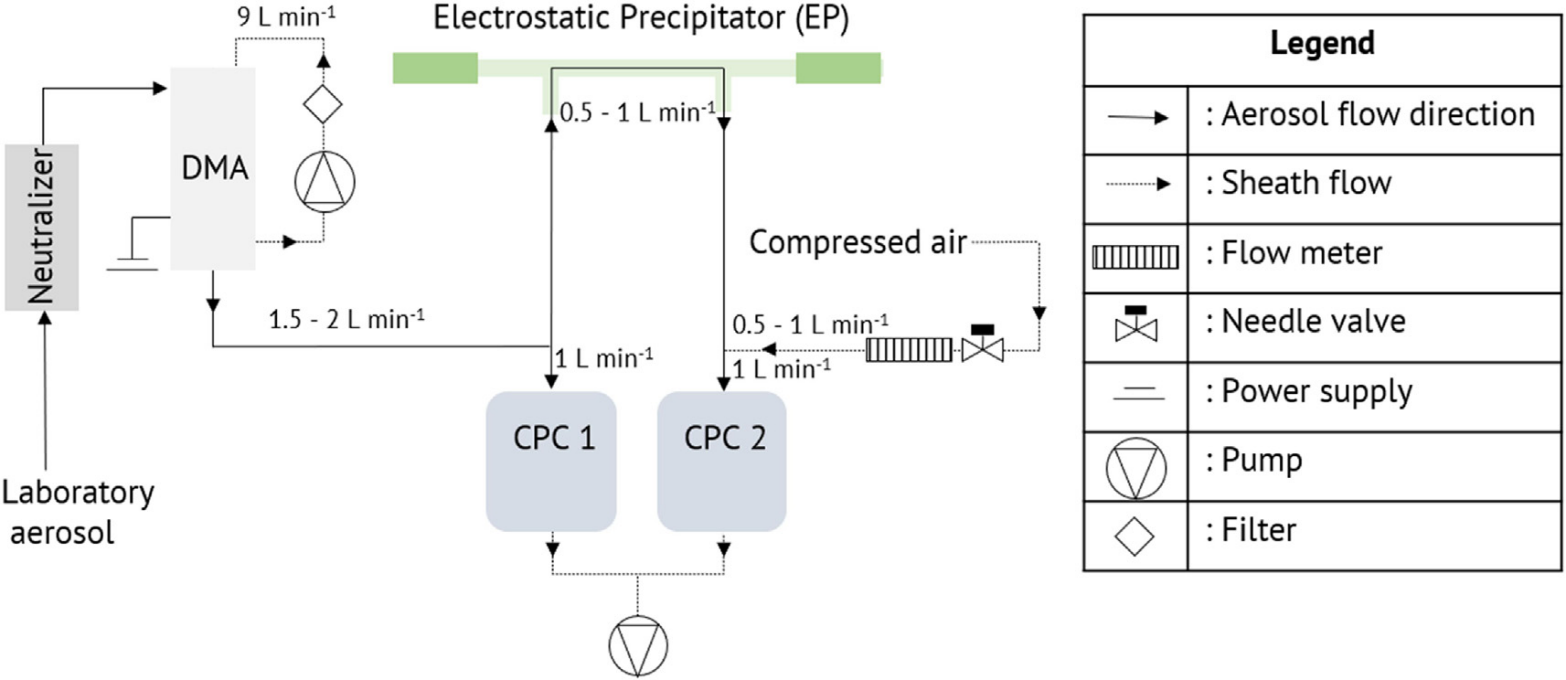
Schematic view of the transmission experiments.

The transmission efficiency was characterized using experiments. Fig. 11 shows the schematic of the setup. To determine the transfer function of the precipitator, monodispersed aerosol was size selected by the DMA and fed through the system while varying the EP voltage. Fraction transmitted was monitored via particle counters on the inlet and outlet. Observations were made at multiple flow rates and different particle mobility diameters selected by DMA. Ambient particle concentration drawn from the lab air was found to be stable enough for the time scales of the experiment. Aerosols (range between 50 nm and 150 nm) are routed through the neutralizer (^210^Po) prior to entering a high-flow DMA [37] that is set up with a recirculating sheath flow loop of 9 L min^−1^. The DMA is operated at a constant voltage (negative polarity, Spellmann SLN10, Hauppauge, NY, USA) to classify particles by mobility (*z*_DMA_). The monodisperse flow is split between a condensation particle counter (CPC, TSI Model 3771) operated at 1 L min^−1^ and the EP. Note that the EP was operated with a power supply of opposite polarity than the DMA, resulting in a collection of the particles on the outer wall of the EP. The choice of positive polarity power supply was dictated by the ability to control low voltages. The choice of positive polarity power supply was dictated by the ability to control low voltages. The control logic of the Ultravolt units is 0–5 V for lowest-to highest voltage for positive polarity power supplies and 5–0 V for lowest-to-highest voltage. Since the DAC system is limited to a maximum 4.095 V signal, the negative polarity power supply cannot be as easily controlled to generate low voltages needed for the transmission experiments.

**Fig. A2:**
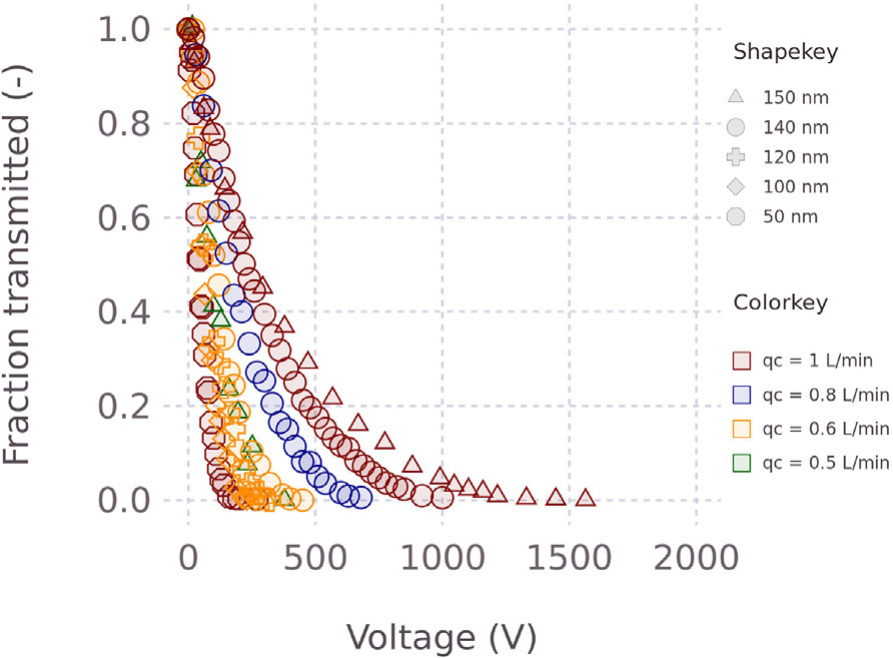
The voltage range for the experiment until the fraction transmitted becomes near zero.

The outlet of the EP is connected to a second CPC also operated at 1 L min^−1^ flow rate. Optionally, particle-free dilution air (Teledyne Model 701, Teledyne Inc., Thousand Oaks, California, U.S.A.) is added between the outlet of the EP and CPC 2. Adding dilution air reduces the flow rate through the EP and alters the sheath-to-sample flow rate of the DMA. The ratio of CPC 2 (corrected for dilution) and CPC 1 concentration provides a measure of the transmission efficiency. Measurements with the unpowered EP were used to correct for the offset between the CPCs, which was < 10% and consistent with the manufacturer's specified accuracy of the instruments. Transmission efficiency was characterized by setting a combination of voltages to the DMA and EP followed by integrating counts for 4 min. The zero transmission for *z*_DMA_/*z*^s^ corresponds to the voltage value where the transmission becomes near zero (see Fig. A2 in the Appendix).

The mean ratio of the two CPCs was computed for a 4 min time segment. Error propagation was used to estimate the 95% confidence interval of the mean for each ratio. Experiments were performed for flow rates of 1, 0.8, 0.6, and 0.5 L min^−1^ flow rate through the EP. The critical *z*^s^ were computed according to Equation (1). However, the length was adjusted to an effective length, where the effective length was adjusted such that transmission for *z*_DMA_ = *z*^s^ was zero.

### Results and discussion

The derived effective lengths are 0.13 m, 0.15 m, 0.208 m, and 0.215 m and increase with decreasing flow rate. The notion of an effective length is qualitatively supported by the CFD simulations, which show significant entrance and exit distortions. The increasing effective length with decreasing flow rate is expected because the entrance length scales with the inlet Reynolds number. Higher flow rates result in longer entrance length and thus shorter effective length. The entrance length for laminar flow in a pipe scales linearly with the Reynolds number [38]. The figures for Reynolds number versus flow rate and Reynolds number versus effective lengths (12′- derived effective lengths) are given in the appendix as a Fig. A4 and Fig. A5.

**Fig. 12:**
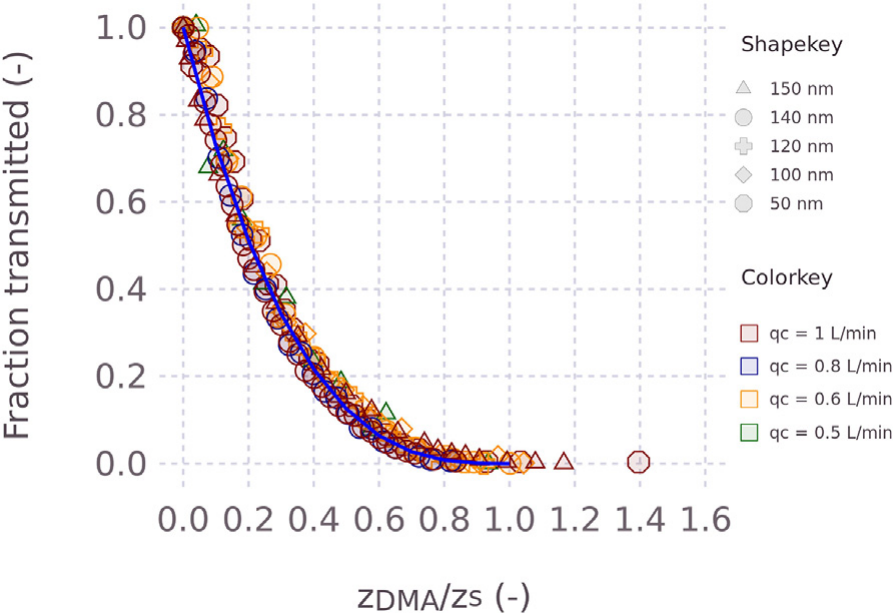
Observed transmission efficiency of different particle mobility diameters (shapes) and flow rates (color). The solid blue line corresponds to the model $T={\left(1-z_{\mathrm{DMA}}/z^{s}\right)}^{3}$. (For interpretation of the references to color in this figure legend, the reader is referred to the web version of this article.)

**Fig. A3:**
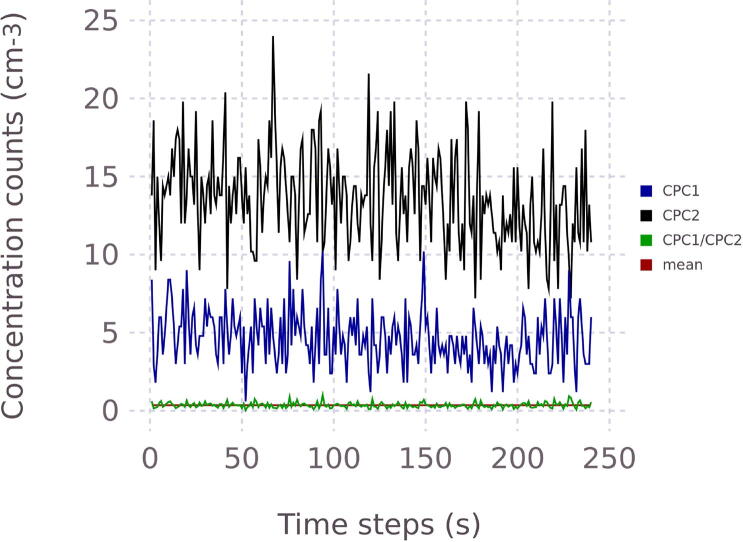
Time series for CPC1 and CPC2 data for 4 min scan of an applied voltage of 20 V. The data correspond to 140 nm particles of 0.6 L min^−1^. Black lines represent concentration as count entering the EP and blue lines show the concentrations as count exiting the EP after an applied voltage for 4 min. The time series of the ratio value of each CPC is given in a green line and the mean value is shown with a red line. The time-series data example for the 2 CPCs is given in Fig. A3 for 140 nm particles of 0.6 L min^−1^. The CPC counts for an applied voltage are given in blue and black lines. The average of the CPCs ratio values is given in green lines with a mean value (red line). (For interpretation of the references to color in this figure legend, the reader is referred to the web version of this article.)

**Fig. A4:**
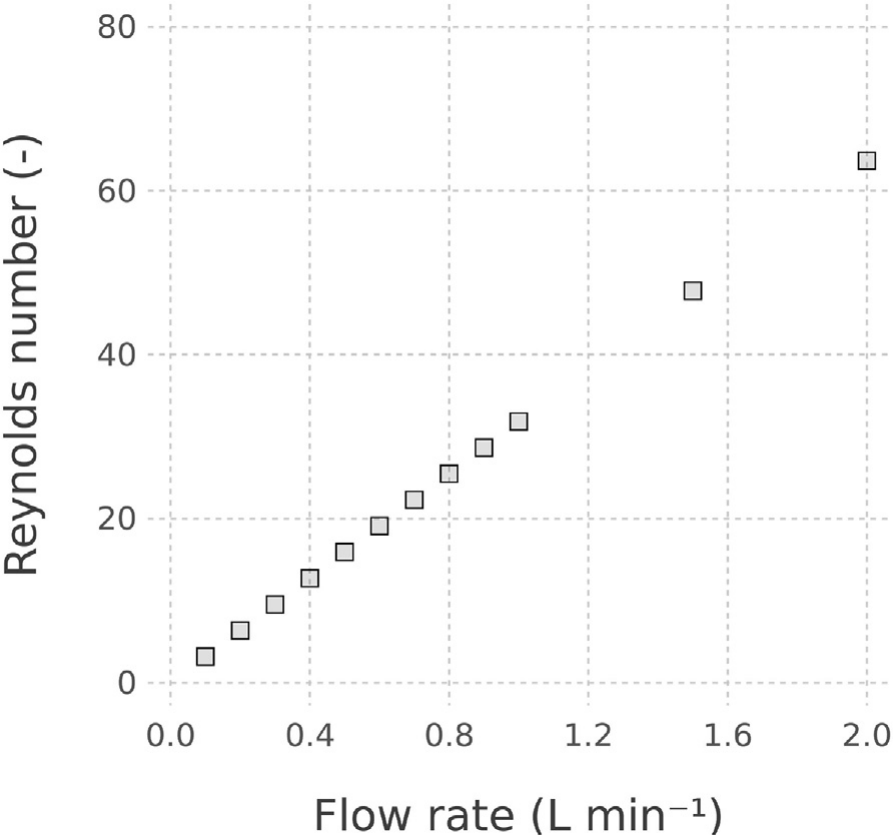
Reynolds number versus flow rate within the EP.

**Fig. A5:**
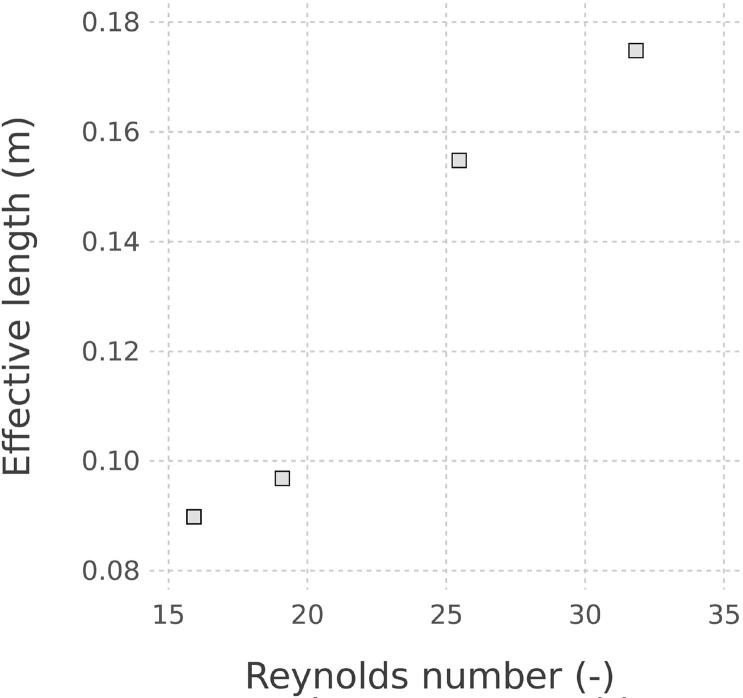
Reynolds number versus effective length(m) for the EP.

Fig. 12 summarizes the transmission efficiency experiments (see Fig. A3 for the transmission of particles in the EP according to the CPCs), which comprise a range of flow rates and mobility diameters. Error bars are omitted from the figure for clarity. The average 95% confidence interval of the derived ratio was ± 0.061. After adjustment for the effective length, the data collapse onto a single transfer function that is described by the empirical formula $T={\left(1-z_{\mathrm{DMA}}/z^{s}\right)}^{3}$. The observations show that the transmission efficiency is significantly smaller than the predicted value for the ideal EP (Fig. 8), even after adjustment for the effective length. The exact reason for the deviation is not known, but particles trapped in the dead volumes to the left of the inlet and to the right of the outlet are a plausible explanation. Despite these non-idealities, the transfer function is reproducible. However, the transfer function should be verified experimentally for each instrument and flow rate rather than relying on the empirical model. In most cases, the purpose of the EP is to remove all charged particles of certain mobility. For this application, the precise transfer function is not relevant and only the value of *z*^s^ is important.

**Fig. 13:**
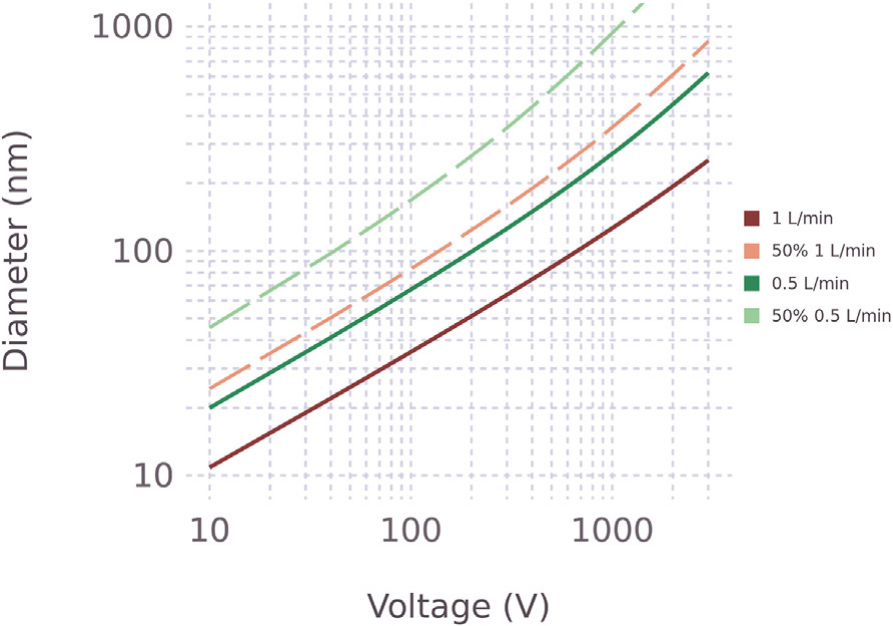
Relationship between voltage and diameter in the EP. Solid lines represent the size where particles are removed to 100%. Dashed lines correspond to a 50% removal efficiency. Calculations are based on Eq. (1), $T={\left(1-z_{\mathrm{DMA}}/z^{s}\right)}^{3}$, and the effective length associated with the flow rate.

The largest recommended voltage applied to the EP for the selected configuration is 3 kV. For larger voltages arcing may occur. Fig. 13 summarizes the voltage required to remove 50% or 100% of singly charged particles as a function of mobility diameter, calculated using *z*^s^ based on the observed effective length. Values are tabulated in Table A1. At 0.5 L min^−1^ and 1 L min^−1^ flow rate, for this geometry of EP, the largest diameters of singly-charged spherical particles that can be removed with 100% efficiency are 624 nm and 253 nm, respectively. The expression *T* = (1- z_DMA_/z_s_)^3^ can be used to estimate the transmission for particles larger than the cutoff. For example, a 1 μm particle and a 0.5 L min^−1^ flow rate the calculated transmission is 0.0765, based on a critical mobility of 1.8 × 10^9^ m^2^ V^−1^s^−1^ and z_DMA_ of 1.1 × 10^9^ m^2^ V^−1^s^−1^.

The primary use for the EP described here is to remove charged particles in the dual tandem DMA [16], [19], [22], [39]. Traditionally, EPs have been used for estimating the neutral fraction after passage through a bipolar neutralizer. A typical setup generates particles, size-classifies particles in a DMA, passes the particles through a bipolar charger [40], [41], [42], [43] followed by passage through the EP [44], [45], [46], [47]. The EP here is suitable for this type of experiment for particles with *D* < ∼600 nm. Another potential application is the purification of air in an indoor environment. However, since the EP only removes charged particles, an aerosol charging device must be used upstream. Unipolar aerosol chargers generally charge 100% of particles with one or multiple charges for *D* > ∼20 nm [48]. Unipolar charging can impart > 10 charges for particle *D* > 100 nm. Thus, the EP here could be used for removal of all particles > 20 nm at a higher flow rate than characterized here. The exact flow rate is unclear due to the effects of turbulence and entrance length in the EP, and flow rate limitations of a hypothetical unipolar aerosol charger. Assuming a flow rate of 2 L min^−1^, it would require 500 s to effectively filter 1 m^−3^ of air. High performance filters are likely a more efficient and more cost-effective method to purify indoor air.

• In this study, we describe and characterize a low-cost design of an electrostatic precipitator. The largest diameters for 100% removal efficiency of singly charged particles are 253 nm and 624 nm for 1 and 0.5 L min^−1^ flow rates, respectively for the given geometry of the EP. The upper size cut can be changed by changing the length of the tube. The total accommodated flow can be changed by scaling the tube dimensions.
• The transfer function for the transmitted particles differs from the ideal precipitator. Even though the transfer function is non-ideal, it is unique for a range of flow conditions. We suspect that the largest source of deviation from the model is due to the variable effective length of the precipitator, and deviation from the theoretically expected transmission efficiency at *z* < *z*^s^. Non-idealities are attributed to the distortion of the flow at the entrance and exit points of the device. The observed non-idealities are qualitatively consistent with computational fluid dynamics simulations of the flow profile.
• The main focus of this study was to design an open-hardware electrostatic precipitator to completely remove charged particles from a stream. The design is versatile, with components such as the fittings and the power‐supply box of the electrostatic precipitator able to be reused in other projects. The power supply box can be used in other common aerosol science applications, including unipolar corona charging [49] or electrospray aerosol generation [50].
• The high-voltage power supply described here was used because we use the same units to power many of our scanning mobility particle sizer (SMPS) systems [51]. Indeed, the power supply box can be used for SMPS data acquisition and control if a USB-to-serial adapter is added to read the concentration from the condensation particle counter. Without the high-voltage power supply (the estimated cost is less than $300), the setup can function as a generic low-cost data acquisition system with GUI (graphical user interface) control.

Additional cost savings are possible if the objective is limited to removing charged particles from the stream, without the need to adjust the voltage. For such a system a mechanical on/off switch and lower-cost high voltage power supply are sufficient. For example, we estimate that an on/off power box with a fixed 1 to 3 kV supply line to the EP can be built for ∼$300 using the Q-series modules from XP-power (Allen, Texas, USA). Such a solution would provide a ∼$400 cost savings over the approach and also obviate the need for a single-board computer and software control. Hardware costs can also be reduced further by using cheaper brass fittings or lower quality compression fittings.

Keeping the cost low and the components generic and reusable may provide a gateway to use the design in a classroom setting to teach students about instrument design and development. Since no machining is needed, assembly of the EP is simple enough that non-engineering graduate students can build the device as part of a research project. Thus, this work may help broaden participation in aerosol science and technology.

### Potential design improvements

Several design choices were made ad-hoc or were based on existing equipment.

• For example, the high-voltage connector assembly could be more elegant. We used a NEMA metal box (see Fig. 2, #13) for the EP enclosure. Drilling through the box is difficult and the overall weight is larger than necessary. A corresponding lighter plastic enclosure might be cheaper and easier to work with.
• The power supply box (see Fig. 4, #9) was not tall enough for DIN rail mounting of the Raspberry Pi computer as intended. If the users prefer to assemble our configuration, they might select a smaller box. If they wish to mount the computer vertically, they would need to select a taller box to accommodate the space for the Raspberry Pi power supply. The use of cable zip ties for ground and electrical connectors is not elegant but works and allows for flexibility in the design. Affixing formal connectors would improve the design.

#### CRediT authorship contribution statement

**Sabin Kasparoglu:** Formal analysis, Data curation, Validation, Writing – original draft, Visualization. **Timothy P. Wright:** Conceptualization, Methodology, Writing – review & editing, Software. **Markus D. Petters:** Supervision, Methodology, Resources, Data curation, Conceptualization, Methodology, Software, Funding acquisition, Project administration, Writing – review & editing.

## Declaration of Competing Interest

The authors declare that they have no known competing financial interests or personal relationships that could have appeared to influence the work reported in this paper.

## Figures and Tables

**Table A1:** Digitized version of Fig 13 in main manuscript. The voltages, diameters, and % 50 particle diameters for 2 flow rates of 0.5 and 1 L min^−1^ are given for spherical particles in the EP.

	1 L min^−1^		0.5 L min^−1^	
V (Volt)	D (nm)	50%D (nm)	D (nm)	50%D (nm)
10	10.86476	24.34499	19.99626	45.52782
40.20202	22.10593	50.52408	41.22762	97.73265
70.40404	29.54059	68.45689	55.60197	135.6928
100.6061	35.5952	83.4524	67.51588	168.8782
130.8081	40.87097	96.81874	78.05513	199.5739
161.0101	45.62977	109.1228	87.69123	228.7472
191.2121	50.01371	120.6705	96.67929	256.9058
221.4141	54.11008	131.6488	105.1756	284.3496
251.6162	57.97705	142.1817	113.2838	311.2698
281.8182	61.65567	152.3559	121.077	337.7949
312.0202	65.17618	162.2351	128.6089	364.0145
342.2222	68.56164	171.8674	135.92	389.9928
372.4242	71.83005	181.2902	143.0422	415.7773
402.6263	74.99583	190.5332	150.0007	441.4032
432.8283	78.07073	199.6203	156.8159	466.8976
463.0303	81.06446	208.5711	163.5048	492.2814
493.2323	83.98515	217.402	170.0815	517.5708
523.4343	86.83971	226.1267	176.5579	542.7786
553.6364	89.63405	234.7568	182.944	567.9154
583.8384	92.37327	243.3023	189.2487	592.9894
614.0404	95.06183	251.7716	195.4794	618.0076
644.2424	97.70363	260.1723	201.6429	642.9756
674.4444	100.3021	268.5108	207.7447	667.8981
704.6465	102.8604	276.7928	213.7902	692.7794
734.8485	105.3811	285.0232	219.7837	717.6227
765.0505	107.8667	293.2066	225.7294	742.4312
795.2525	110.3195	301.3468	231.6308	767.2073
825.4545	112.7414	309.4473	237.4913	791.9534
855.6566	115.1343	317.5113	243.3137	816.6715
885.8586	117.4998	325.5416	249.1006	841.3635
916.0606	119.8394	333.5408	254.8546	866.031
946.2626	122.1545	341.511	260.5778	890.6754
976.4646	124.4464	349.4545	266.2722	915.2981
1006.667	126.7162	357.373	271.9396	939.9003
1036.869	128.9651	365.2683	277.5817	964.4833
1067.071	131.194	373.1418	283.2	989.048
1097.273	133.4039	380.9951	288.7959	1013.595
1127.475	135.5956	388.8294	294.3708	1038.126
1157.677	137.77	396.6459	299.9259	1062.642
1187.879	139.9278	404.4456	305.4622	1087.143
1218.081	142.0698	412.2297	310.9809	1111.629
1248.283	144.1965	419.9989	316.4828	1136.103
1278.485	146.3086	427.7541	321.9688	1160.563
1308.687	148.4067	435.4961	327.4399	1185.012
1338.889	150.4913	443.2256	332.8967	1209.449
1369.091	152.563	450.9433	338.3399	1233.875
1399.293	154.6222	458.6498	343.7704	1258.291
1429.495	156.6694	466.3457	349.1885	1282.697
1459.697	158.7051	474.0314	354.5951	1307.093
1489.899	160.7296	481.7074	359.9905	1331.48
1520.101	162.7434	489.3744	365.3754	1355.859
1550.303	164.7468	497.0325	370.7502	1380.229
1580.505	166.7402	504.6823	376.1154	1404.591
1610.707	168.7239	512.3241	381.4713	1428.946
1640.909	170.6982	519.9582	386.8185	1453.293
1671.111	172.6634	527.585	392.1572	1477.634
1701.313	174.6199	535.2048	397.4878	1501.967
1731.515	176.5679	542.8178	402.8107	1526.295
1761.717	178.5076	550.4243	408.1262	1550.616
1791.919	180.4394	558.0246	413.4346	1574.931
1822.121	182.3634	565.6189	418.7361	1599.241
1852.323	184.2799	573.2074	424.0311	1623.545
1882.525	186.1892	580.7903	429.3197	1647.844
1912.727	188.0913	588.3679	434.6023	1672.138
1942.929	189.9866	595.9402	439.879	1696.427
1973.131	191.8752	603.5075	445.1501	1720.711
2003.333	193.7573	611.0699	450.4158	1744.991
2033.535	195.6332	618.6276	455.6762	1769.267
2063.737	197.5028	626.1808	460.9315	1793.538
2093.939	199.3666	633.7295	466.182	1817.806
2124.141	201.2245	641.2739	471.4278	1842.069
2154.343	203.0767	648.8142	476.6689	1866.329
2184.545	204.9235	656.3504	481.9057	1890.586
2214.747	206.7648	663.8826	487.1382	1914.839
2244.949	208.601	671.4111	492.3666	1939.088
2275.152	210.4321	678.9358	497.591	1963.335
2305.354	212.2582	686.4568	502.8115	1987.578
2335.556	214.0794	693.9744	508.0282	2011.818
2365.758	215.8959	701.4885	513.2413	2036.055
2395.96	217.7079	708.9992	518.4508	2060.29
2426.162	219.5153	716.5066	523.6569	2084.522
2456.364	221.3184	724.0109	528.8597	2108.751
2486.566	223.1171	731.512	534.0592	2132.977
2516.768	224.9117	739.0101	539.2555	2157.201
2546.97	226.7022	746.5052	544.4488	2181.423
2577.172	228.4887	753.9973	549.6391	2205.643
2607.374	230.2713	761.4867	554.8264	2229.86
2637.576	232.0501	768.9732	560.0109	2254.075
2667.778	233.8252	776.457	565.1927	2278.287
2697.98	235.5966	783.9382	570.3717	2302.498
2728.182	237.3645	791.4167	575.5481	2326.707
2758.384	239.1289	798.8927	580.7219	2350.914
2788.586	240.8899	806.3661	585.8933	2375.119
2818.788	242.6475	813.8371	591.0621	2399.322
2848.99	244.4019	821.3057	596.2286	2423.523
2879.192	246.1531	828.772	601.3928	2447.723
2909.394	247.9012	836.2359	606.5546	2471.921
2939.596	249.6462	843.6975	611.7142	2496.117
2969.798	251.3882	851.157	616.8717	2520.312
3000	253.1273	858.6142	622.0269	2544.505

## Appendix

Fig. A1 shows the high voltage connectors used in our system. Fig. A2 shows the applied voltage through the precipitator to attain a fraction transmitted near zero. The zero transmission for *z*_DMA_/*z*^s^ corresponds to voltage values where the transmission becomes near zero. The experiments correspond to Fig. 12 in the main manuscript. In Fig. A3, the time series for CPC1 and CPC2 data for 4 min scan of an applied voltage of 20 V. Fig. A4 and Fig. A5 show the Reynolds number versus flow rate and Reynolds number versus effective lengths  (12′- derived effective lengths), respectively.
